# N-Terminal Prolactin-Derived Fragments, Vasoinhibins, Are Proapoptoptic and Antiproliferative in the Anterior Pituitary

**DOI:** 10.1371/journal.pone.0021806

**Published:** 2011-07-07

**Authors:** Jimena Ferraris, Daniela Betiana Radl, Sandra Zárate, Gabriela Jaita, Guadalupe Eijo, Verónica Zaldivar, Carmen Clapp, Adriana Seilicovich, Daniel Pisera

**Affiliations:** 1 Instituto de Investigaciones en Reproducción, Facultad de Medicina, Universidad de Buenos Aires, Buenos Aires, Argentina; 2 Instituto de Neurobiología, Universidad Nacional Autónoma de México, Juriquilla, México; New Mexico State University, United States of America

## Abstract

The anterior pituitary is under a constant cell turnover modulated by gonadal steroids. In the rat, an increase in the rate of apoptosis occurs at proestrus whereas a peak of proliferation takes place at estrus. At proestrus, concomitant with the maximum rate of apoptosis, a peak in circulating levels of prolactin is observed. Prolactin can be cleaved to different N-terminal fragments, vasoinhibins, which are proapoptotic and antiproliferative factors for endothelial cells. It was reported that a 16 kDa vasoinhibin is produced in the rat anterior pituitary by cathepsin D. In the present study we investigated the anterior pituitary production of N-terminal prolactin-derived fragments along the estrous cycle and the involvement of estrogens in this process. In addition, we studied the effects of a recombinant vasoinhibin, 16 kDa prolactin, on anterior pituitary apoptosis and proliferation. We observed by Western Blot that N-terminal prolactin-derived fragments production in the anterior pituitary was higher at proestrus with respect to diestrus and that the content and release of these prolactin forms from anterior pituitary cells in culture were increased by estradiol. A recombinant preparation of 16 kDa prolactin induced apoptosis (determined by TUNEL assay and flow cytometry) of cultured anterior pituitary cells and lactotropes from ovariectomized rats only in the presence of estradiol, as previously reported for other proapoptotic factors in the anterior pituitary. In addition, 16 kDa prolactin decreased forskolin-induced proliferation (evaluated by BrdU incorporation) of rat total anterior pituitary cells and lactotropes in culture and decreased the proportion of cells in S-phase of the cell cycle (determined by flow cytometry). In conclusion, our study indicates that the anterior pituitary production of 16 kDa prolactin is variable along the estrous cycle and increased by estrogens. The antiproliferative and estradiol-dependent proapoptotic actions of this vasoinhibin may be involved in the control of anterior pituitary cell renewal.

## Introduction

In sexually mature female mammals, hypothalamic, pituitary and ovarian hormones have particular secreting profiles that define the endocrine environment of each stage of the estrous cycle. In the rat anterior pituitary, cell renewal is also a cyclic phenomenon. In each estrous cycle a peak of proliferation occurs at estrus whereas the highest rate of apoptosis is observed during proestrus [1], [2]. The cell turnover that takes place in the rat anterior pituitary is a highly regulated process in which several factors were demonstrated to participate. Estradiol, by the induction of apoptosis per se or by sensitizing cells to different proapoptotic factors such as TNF-α, FasL and dopamine, plays a key role in anterior pituitary gland remodeling [3]–[7].

Prolactin plasma levels are relatively constant along the estrous cycle, except during proestrus, when an abrupt increase in its secretion takes place, mainly due to the concomitant high circulating levels of estrogens [8]. Apart from the 23 kDa native protein, prolactin is present as several molecular forms that include phosphorylated, glycosylated, and lower molecular weight proteins [9]. Enzymatic cleavage of 23 kDa prolactin produces 14 to 18 kDa amino-terminal fragments with antiangiogenic effects, known as vasoinhibins [10]. 16 KDa prolactin has been strongly related to antiproliferative and proapoptotic actions in endothelial cells inducing cell cycle arrest [11], caspase activation [12], [13], modulation of Bcl-2 protein family [13] and NFκB activation [13].

It has been shown that a 16 kDa amino terminal fragment is produced in the anterior pituitary gland by cathepsin D [14]. Like prolactin expression, 16 kDa prolactin production in this gland could also be a hormone-regulated process. In fact, Souto et al suggested that its rate of production could be gender-dependent [14].

In this work, we investigated the production of anterior pituitary N-terminal prolactin fragments along the estrous cycle and the effect of estradiol on their content and release. Because of vasoinhibins reported actions on endothelial cells [10], we also studied whether 16 kDa prolactin is involved in the control of anterior pituitary cell renewal.

## Materials and Methods

### Drugs

All drugs, media and supplements were obtained from Invitrogen (Carlsbad, CA, USA), except Dulbecco's modified Eagle's medium (DMEM), EDTA, bovine serum albumin (BSA), 17β-estradiol, bromodeoxyuridine (BrdU), protease inhibition kit, normal donkey and sheep sera, anti β-actin antibody, deoxyribonuclease type I, forskolin (Sigma, St. Louis, MO, USA), fetal bovine serum (Natocor, Argentina), all terminal deoxynucleotidyl transferase-mediated deoxyuridine triphosphate nick end-labeling (TUNEL) reagents (Roche Molecular Biochemicals, Mannheim, Germany), guinea pig rat prolactin antiserum, anti-recombinant rat prolactin antiserum (rrPRL) (Dr. A. Parlow, National Hormone and Pituitary Program, Torrance, CA, USA), anti-BrdU (BD Bioscience) anti-guinea pig rhodamine-conjugated secondary antibody, anti-mouse fluorescein isothiocyanate (FITC) conjugated secondary antibody, streptavidin horseradish peroxidase (HRP) conjugated anti-rabbit antibody (Chemicon International, Temecula, CA, USA) and the materials indicated below.

### Animals

Adult female Wistar rats were kept in controlled conditions of light (12-hour light-dark cycles) and temperature (20–25°C). Rats were fed standard lab chow and water ad libitum and kept in accordance with the NIH Guide for the Care and Use of Laboratory Animals. All the procedures were in compliance with the Ethical Committee of the School of Medicine, University of Buenos Aires (Res. (CD) N° 2831/10). Rats were ovariectomized (OVX) under ketamine (100 mg/kg, i.p.) and xylazine (10 mg/kg, i.p.) anesthesia 2 weeks before the experiments. Anterior pituitary glands were removed within minutes after decapitation and processed for primary culture. In addition, cycling female rats were monitored by daily vaginal smears. Rats with three or more normal consecutive 4–5 day estrous cycles were killed between 12:30 pm and 1 pm of diestrus I or proestrus. Anterior pituitaries from cycling rats were processed for protein extraction.

### Anterior pituitary primary culture

A pool of anterior pituitaries from 3–4 OVX rats was used for each culture. The glands were washed with Dulbecco's Modified Eagle Medium supplemented with 10 µl/ml MEM amino acids, 2 mM glutamine, 5.6 µg/ml amphotericin B, 25 µg/ml gentamicin (DMEM) and 3 mg/ml BSA. Then, anterior pituitaries were cut into small fragments. Sliced fragments were dispersed enzymatically by successive incubations in DMEM-BSA containing 0.75% trypsin, 10% fetal calf serum previously treated with 0.025% dextran-0.25% charcoal to remove steroids (FCS), and 45 U/µl deoxyribonuclease type I (DNAse). Finally, the cells were dispersed by extrusion through a Pasteur pipette in Krebs buffer without Ca^2+^ and Mg^2+^. Dispersed cells were washed and resuspended in DMEM with 10% FCS. Cell viability as assessed by trypan blue exclusion was over 90%. Dispersed cells were seeded onto 24-well tissue culture plates for BrdU incorporation detection, TUNEL assay or culture media analysis (1.5–2×10^5^ cells/ml/well), for flow cytometry (FACS) (3×10^5^ cells/ml/well) and for protein extraction (1×10^6^ cells/ml/well). Cells were cultured for 24 h in DMEM with 10% FCS (DMEM-FCS) and then incubated for 24 h in DMEM-FCS containing ethanol (1 µl/l, VEH) or 17β-estradiol (10^−9^ M, E2) for the detection of N-terminal prolactin fragments in anterior pituitary cells and culture media. For apoptosis and cell cycle evaluation experiments, cells were incubated for 19 h in DMEM without FCS in the presence of VEH or E2. Then, cells were incubated with the same fresh media for 1 h and then incubated for further 4 h in the same media with or without 16 kDa prolactin (16 kDa PRL, 10 nM) [15]. Since BrdU incorporation (3 h) was not detected in cells cultured in DMEM-FCS (data not shown), for proliferation studies cells were incubated in the presence of forskolin (1 µM, FSK), which induces anterior pituitary cell proliferation in vitro [16]. In some experiments cells were incubated for 19 h with FSK and VEH. Then, cells were incubated with the same fresh media for 1 h and then for further 4 h in the same media with or without 16 kDa PRL (10 nM). In other experiments, cells were incubated for 19 h with E2. Then, cells were incubated with FSK and E2 for 1 h and then, for further 4 h in the same media with or without 16 kDa PRL (10 nM). Three hours before fixation, BrdU (200 µM) was added to the culture media. Recombinant 16 kDa PRL (16 kDa PRL) was obtained as previously described [17].

### Western blot

Total proteins were extracted from anterior pituitary glands or from cultured anterior pituitary cells with lysis buffer containing 250 mM NaCl, 5 mM MgCl_2_, 50 mM NaF, 1 mM dithiothreitol, 1% Igepal, 0.02% sodium azide, 0.1% sodium dodecyl sulphate (SDS), in 50 mM Tris-HCl pH 7.4 and a protease inhibitor kit (1∶100). Following homogenization and centrifugation at 16000 rcf for 30 min, the supernatant was used for the immunoblot assay. The protein concentration of each sample was determined by the Bradford protein assay (BioRad Laboratories, CA, USA). Thirty µg of total proteins or 15 µl of culture media from anterior pituitary cells in culture were size-fractionated in 15% SDS-polyacrylamide gel and then electrotransferred to polyvinyl difluoride (PVDF) membranes. Blots were incubated for 90 min in 5% nonfat dry milk-TBS-0.1% Tween 20 at room temperature and incubated overnight with the appropriate primary antibody in the same buffer at 4°C. The primary antibodies used were: anti-rat recombinant PRL (anti-rrPRL 1∶10000 to 1∶25000) and anti β-actin (1∶5000). This was followed by 1 h incubation with the corresponding secondary antibody conjugated with HRP. Proteins incubated with buffer without primary antibody were used as negative controls. Immunoreactivity was detected by enhanced chemoluminescence (Productos Bio-Lógicos, Argentina). Chemoluminiscence was detected by chemoluminiscence imaging system (G Box Chemi HR16, Syngene) and bands were quantified using Gene Tools software (Syngene). Intensity data were normalized with respect to the corresponding β-actin blot. Data were expressed as relative increment to respective controls.

### TUNEL, BrdU incorporation and Immunocytochemistry

After the incubation period, cultured cells were fixed with 4% paraformaldehyde for 10 min and permeabilized by microwave irradiation. For TUNEL assay, DNA strand breaks were labeled with digoxigenin-deoxyuridine triphosphate using terminal deoxynucleotidyl transferase (0.18 U/µl) according to the manufacturer's protocol. After incubation with 10% normal donkey serum and 10% normal sheep serum in PBS for 90 min, cells were incubated for 1 h with guinea pig rat prolactin antiserum (1∶1500). Then, slides were incubated with antidigoxygenin-fluorescein antibody (1∶10) to detect incorporation of nucleotides into the 3′-OH end of damaged DNA and rhodamine-conjugated anti-guinea pig secondary antibody (1∶200) in the same buffer. For BrdU incorporation assay, the slides were incubated 30 min with DNAse (45 U/µl) at 37°C to break DNA strands and to allow anti-BrdU antibody to interact with incorporated BrdU. Then, cells were blocked 90 min with 10% horse serum in PBS- 0.2% Triton and then incubated with mouse anti-BrdU antibody (1∶200) overnight. The next day, slides were incubated with a FITC-conjugated anti-mouse secondary antibody (1∶200). After incubation with 10% normal donkey serum in PBS-0.2% Triton for 90 min, cells were incubated for 1 h with guinea pig rat prolactin antiserum (1∶1500) followed by 1 h incubation with rhodamine-conjugated anti-guinea pig secondary antibody (1∶200). Control slides were incubated with normal serum instead of primary antibodies. Slides were mounted with Vectashield (Vector Laboratories, Inc., Burlingame, CA, USA) containing 4, 6 diamidino-2-phenylindoledihydrochloride (DAPI) for DNA staining and visualized in a fluorescent light microscope (Axiophot; Carl Zeiss, Jena, Germany). The percentage of lactotropes determined by immunocytochemistry represented 25–40% of the population of anterior pituitary cells. The percentage of apoptotic (TUNEL-positive) or proliferating (BrdU-positive) anterior pituitary cells was calculated as (TUNEL-positive or BrdU-positive cells/total anterior pituitary cells)×100; and the percentage of apoptotic or proliferating lactotropes was calculated as (TUNEL-positive or BrdU-positive prolactin-immunoreactive cells/total prolactin-immunoreactive cells)×100.

### Flow cytometry

Cultured anterior pituitary cells from OVX rats were harvested with 0.05% trypsin-EDTA. Cells of each well were washed with PBS, resuspended in 300 µl of PBS-0.1% BSA and added for fixation into 5 ml of 70% ice-cold methanol by gentle vortexing. Cells were fixed for 30 min at −20°C. Then, 5 ml of PBS-0.1% BSA were added to each tube. After centrifugation, DNA was stained with propidium iodide (PI, 50 µg/ml) in PBS containing ribonuclease (10 µg/ml) at 37°C for 20 min. Fluorescence intensity of ≥6000 gated cells/tube was analyzed by flow cytometry using a FACScan (Becton Dickinson). Cells with a PI staining intensity lower than the G0/G1 peak were considered hypodiploid. Analysis of hypodiploid or S-phase DNA content was performed using WinMDI 98 and Cylcherd 1.2 software.

### Statistical Analysis

The number of TUNEL-positive cells and the number of BrdU positive-cells were determined in slides from independent experiments. Results were expressed as the percentage ±95% confidence interval (CI) of TUNEL-positive cells or BrdU-positive cells of the total number of cells counted in each specific condition. Differences between proportions were analyzed by χ^2^. The percentage of hypodiploid cells or cells in S-phase of the cell cycle were expressed as mean ± SE and analyzed by unpaired Student's t test. Western blot data were expressed as mean ± SE and analyzed by unpaired Student's t test. Differences were considered significant if p<0.05. Statistical analysis was performed using GraphPad Prism 4 software.

## Results

### N-terminal PRL fragments content in the anterior pituitary along the estrous cycle

**Figure 1:**
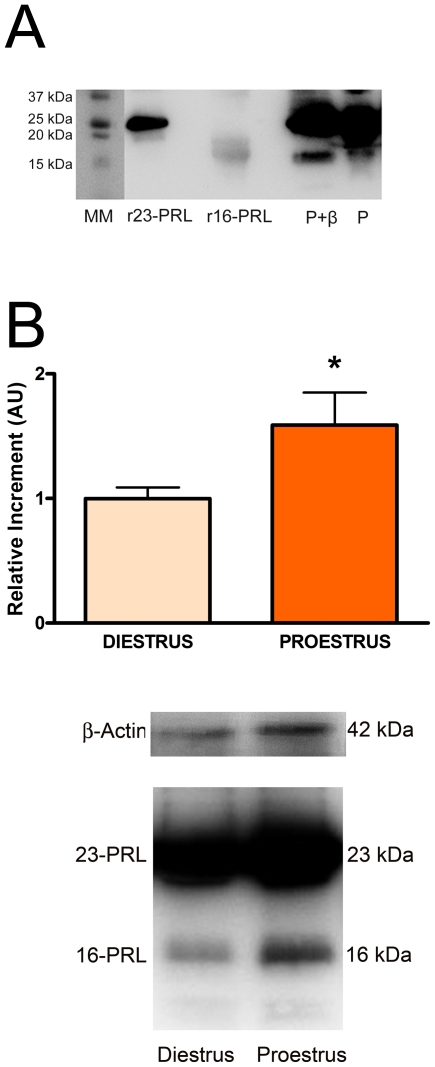
N-terminal PRL fragments content in the anterior pituitary varies along the estrous cycle. A: Recombinant 23 kDa PRL (r23-PRL), recombinant 16 kDa prolactin (r16-PRL) or pituitary protein extracts treated with β-mercaptoethanol (P+β, reducing conditions) or without β-mercaptoethanol (P, non-reducing conditions) were incubated with anti-recombinant rat PRL antibody (anti rrPRL, 1∶25000). The antibody recognizes both PRL forms. B: Anterior pituitaries from rats euthanized at diestrus I or proestrus were processed for western blot. Upper panel: Each column represents the mean ± SE of the relative increment of N-terminal PRL fragments content with respect to diestrus I. Data of each column were normalized to β-actin expression (n = 4–7 animals per group). *p<0.01 vs. diestrus I, Student's t test. Lower panel: Representative blot of pituitary proteins from rats euthanized at diestrus I or proestrus.

To evaluate the ability of the anti-rrPRL to bind to 23 kDa PRL and N-terminal PRL fragments present in the anterior pituitary [14], we tested its reactivity against recombinant 16 kDa PRL, recombinant 23 kDa PRL and protein extracts obtained from anterior pituitaries of female rats by Western blot. As shown in Figure 1A, the antibody recognized 23 kDa PRL and 16 kDa N-terminal PRL standards. Additionally, 23 kDa and ≈16 kDa bands were also visible when protein extracts from the anterior pituitary were run under reducing and non-reducing conditions. As previously described by Cruz-Souto et al., a higher intensity in the ≈16 kDa band was observed under reducing conditions [14] (Fig. 1A). The 23 kDa and ≈16 kDa variants were also recognized by an anti-N-terminal PRL fragment antibody as previously described [14] (data not shown).

As circulating PRL levels peak during proestrus, when the rate of anterior pituitary cell apoptosis is the highest, we evaluated if the content of N-terminal PRL fragments in the anterior pituitary changes during the estrous cycle. Protein extracts from anterior pituitaries of female rats euthanized at proestrus or diestrus I were analyzed by western blot using anti-rrPRL. The content of 16 kDa PRL was higher in anterior pituitaries from rats euthanized at proestrus than at diestrus I (Fig. 1B and 1C).

### Production and release of N-terminal PRL fragments by anterior pituitary cells

**Figure 2:**
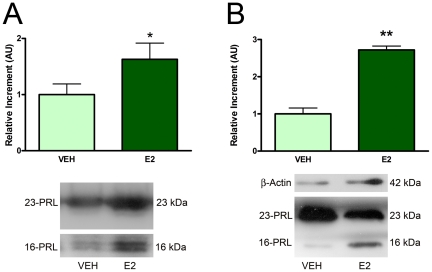
Estradiol increases the secretion and content of N-terminal PRL fragments from anterior pituitary cells in culture. Anterior pituitary cells from OVX rats were incubated in the presence of VEH or E2 for 24 h. A: Culture media and B: cells were obtained and processed for western blot analysis. Each column represents the mean ± SE of the relative increment of N-terminal PRL fragments with respect to VEH. In B, data of each column were normalized to β-actin expression (A, n = 6 wells/group; B, n = 6 wells/group), representative of three independent experiments. *p<0.05, **p<0.01 vs. VEH without E2, Student's t test. Lower panels: Representative blots of media (A) or cells (B) cultured with VEH or E2.

16 kDa PRL was previously shown to be produced by cultured anterior pituitary cells from OVX-E2 treated rats [14]. To evaluate if estrogens are involved in the increase of the content of 16 kDa PRL in the anterior pituitary observed during proestrus, we incubated anterior pituitary cells from OVX rats in the presence or absence of E2. In vitro secretion and cellular content of N-terminal PRL fragments were evaluated by western blot. The presence of E2 induced an increase in both release (Fig. 2A) and content (Fig. 2B) of 16 kDa PRL from anterior pituitary cells in culture.

### 16 kDa PRL and apoptosis of anterior pituitary cells

**Figure 3:**
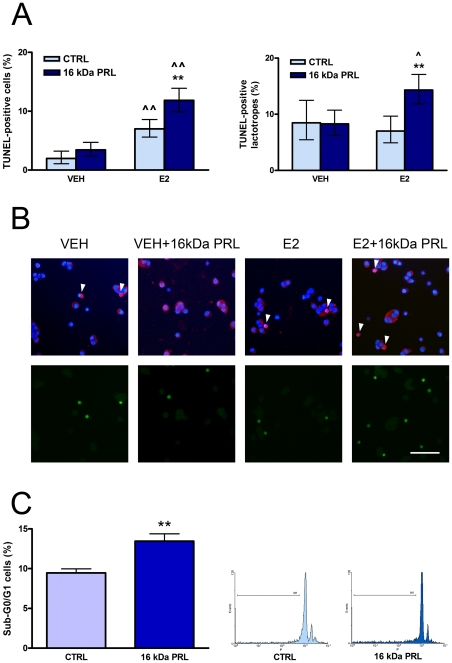
16 kDa PRL induces apoptosis of anterior pituitary cells. A and B: Anterior pituitary cells from OVX rats were incubated with VEH or E2 for 24 h and with or without 16 kDa PRL (10 nM) for the last 4 h. A: Each column represents the percentage ± CI (95%) of TUNEL-positive cells (n≥1500 cells/group, left) or TUNEL-positive lactotropes (n≥300 cells/group, right), representative of four independent experiments. Data were analyzed by χ^2^. **p<0.01 vs. respective control without 16 kDa PRL. ∧∧p<0.01, ∧p<0.05 vs. respective control without E2. B: Representative images of anterior pituitary cells showing immunoreactivity for prolactin (red) counterstained with DAPI (blue, upper panels) and DNA fragmentation determined by TUNEL method (green, lower panels). Arrowheads indicate apoptotic lactotropes. Scale bar: 50 µm. C: Anterior pituitary cells from OVX rats were incubated with E2 for 24 h, and with or without 16 kDa PRL (10 nM) for the last 4 h. The percentage of hypodiploid cells was determined by flow cytometry using PI. Left: Each column represents the mean ± SE of the percentage of sub-G1 cells (n = 3 wells/group), representative of three independent experiments. Data were analyzed by Student's t test. **p<0.01 vs. respective control without 16 kDa PRL. Right: Representative histograms of fluorescence intensity of DNA content of anterior pituitary cells incubated in the presence or absence of 16 kDa PRL.

To test the effect of 16 kDa PRL on anterior pituitary cell apoptosis, primary cultures of anterior pituitary cells from OVX rats were incubated with 16 kDa PRL (10 nM) [18] for 4 h and evaluated by TUNEL. As E2 sensitizes anterior pituitary cells to different proapoptotic stimuli [3]–[6], [18], the apoptotic effect was determined in the presence or absence of E2. As previously described [6], [19], E2 itself induced apoptosis of total anterior pituitary cells. 16 kDa PRL increased the percentage of TUNEL-positive cells and TUNEL-positive lactotropes only in the presence of E2 (Fig. 3A and 3B). To confirm this effect, we evaluated the percentage of hypodiploid cells by flow cytometry. In the presence of E2, 16 kDa PRL increased the percentage of cells in Sub-G0/G1 (Fig. 3C).

### 16 kDa PRL and anterior pituitary cell proliferation

**Figure 4:**
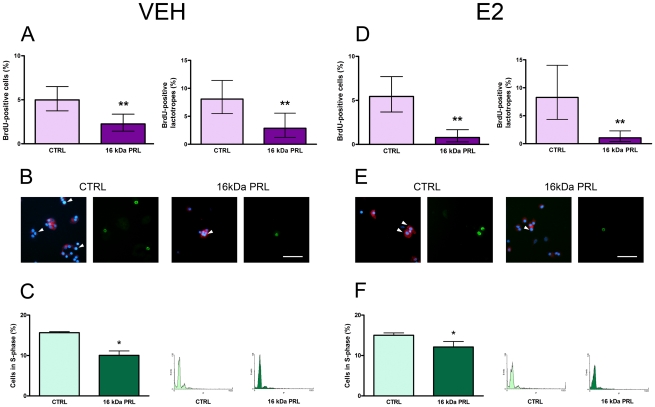
16 kDa PRL inhibits anterior pituitary cell proliferation. A and B: Anterior pituitary cells from OVX rats were incubated for 24 h with VEH and FSK, and with or without 16 kDa PRL (10 nM) for the last 4 h. D and E: Anterior pituitary cells from OVX rats were incubated for 24 h with E2, then for 1 h with E2 and FSK, and for the last 4 h in the same media with or without 16 kDa PRL (10 nM). Proliferation was determined by BrdU incorporation (3 h) and fluorescence microscopy. A and D: Each column represents the percentage ± CI (95%) of BrdU-positive cells (n≥1000 cells/group, left) or BrdU-positive lactotropes (n≥300 cells/group, right), representative of four independent experiments. Data were analyzed by χ^2^. **p<0.01 vs. respective control without 16 kDa PRL. B and E: Representative images of anterior pituitary cells showing immunoreactivity for prolactin (red) counterstained with DAPI (blue) and BrdU (green). Arrowheads indicate proliferating lactotropes. Scale bar: 50 µm. C and F: Anterior pituitary cells in culture were incubated with VEH (C) or E2 (F) for 24 h, and with or without 16 kDa PRL (10 nM) for the last 4 h. Cell cycle was analyzed by flow cytometry using PI. Left: Each column represents the mean ± SE of the percentage of S-phase cells (n≥4 wells/group), representative of three independent experiments. Data were analyzed by Student's t test. *p<0.05 vs. respective control without 16 kDa PRL. Right: Representative histograms of DNA content of anterior pituitary cells incubated in the presence or absence of 16 kDa PRL.

To evaluate the effect of 16 kDa PRL on proliferation of total anterior pituitary cells and lactotropes, primary cultures from OVX rats were incubated in the presence of FSK with or without E2. The effect of 16 kDa PRL (10 nM, 4 h) was evaluated by BrdU incorporation. 16 kDa PRL decreased FSK-induced proliferation in total anterior pituitary cells and lactotropes both in the absence (Fig. 4A and B) and presence (Fig. 4D and E) of E2.

Additionally, we evaluated the effect of 16 kDa PRL on cell cycle progression of anterior pituitary cells by flow cytometry. Anterior pituitary cells from OVX rats were cultured with VEH or E2 and then incubated for 4 h in the absence or presence of 16 kDa PRL (10 nM). Confirming its antiproliferative action observed in BrdU incorporation studies, 16 kDa PRL decreased the percentage of cells in S-phase in the absence (Fig. 4C) or presence (Fig. 4F) of E2.

## Discussion

It has been reported that 16 kDa PRL and other N-terminal PRL-derived fragments have proapoptotic and antiproliferative actions in endothelial cells. Because of its antiangiogenic properties, this group of molecules is known as vasoinhibins [10]. These peptides are produced by proteolytic cleavage from 23 kDa prolactin. Bone morphogenetic protein, matrix metalloproteinases and cathepsin D have been involved in this process [20], [10]. Cathepsin D is an aspartic protease with an optimum pH of 4.5–5.0 for its activity [21], therefore it may be active in lysosomal granules where this pH condition occurs. Cathepsin D can also be secreted to the extracellular compartment [22] where its activity is controversial due to the neutral pH of this environment. In bovine corpora lutea, 23 kDa PRL conversion to 14 and 16 kDa fragments by secreted cathepsin D was reported to be optimal at pH 3 and inhibited at pH 7 [23]. However, it has been shown that mammary gland epithelial cells secrete cathepsin D able to produce PRL-derived 16 kDa products at pH 7 in the extracellular milieu [21], [24]. On the other hand, acidic pH in the extracellular compartment can occur under certain circumstances, such as low oxygen tension that can be found in tumors [25], [26] Also, it was shown that in normal conditions, a pericellular acidic pH can be reached by the activity of the Na+/H+ exchanger and H+/ATPase [24]. In anterior pituitary cells, cathepsin D is present in prolactin secretory granules, and was proposed to be the main protease involved in 16 kDa PRL production, as mice deficient for cathepsin D do not generate vasoinhibins in the anterior pituitary gland [14]. Our present data show the presence of N-terminal PRL fragments in cultured anterior pituitary cells and in the conditioned media as previously reported [10], [27], supporting the hypothesis of intracellular production of 16 kDa PRL and suggesting that the anterior pituitary could be a source for circulating 16 kDa PRL.

In our study we demonstrate that the production of N-terminal PRL fragments in the anterior pituitary gland varies during the estrous cycle, being higher at proestrus than at diestrus. In addition, we show that E2 increases anterior pituitary cell content and release of 16 kDa PRL, suggesting that estrogens are involved in the increase of N-terminal PRL fragments production observed during proestrus. In fact, anterior pituitary cells from OVX-E2 treated rats show a higher content of 16 kDa PRL than cells obtained from male rats [14]. The preovulatory peak of 23 kDa PRL plasma levels observed in the afternoon of proestrus is due to the high circulating levels of E2, which increase anterior pituitary PRL gene expression and PRL release [8]. This hormonal status may also induce a rise in 16 kDa PRL secretion at this stage of the estrous cycle. An increase in a given enzymatic product could be due to an increment in substrate availability and/or an increase in enzymatic activity. In this regard, it was shown that cathepsin D activity in corpora lutea varies along the estrous cycle [23] and that estradiol increases the activity and synthesis of cathepsin D in uterus [28] and in ovarian and breast cancer cells [29]. Hence, both an increase in 23 kDa prolactin and anterior pituitary cathepsin D activity in response to E2 at proestrus could be responsible for the higher levels of 16 kDa PRL found in the anterior pituitary at this stage of the estrous cycle.

Our data show that 16 kDa PRL has a proapoptotic action in anterior pituitary cells and lactotropes. Anterior pituitary cell homeostasis is regulated by circulating levels of gonadals steroids, since a peak of apoptosis occurs during proestrus when estradiol levels are the highest, whereas the maximum proliferation rate takes place at estrus [1], [2]. Estadiol is known to play a key role in this process, and is required to trigger TNF-α, FasL, dopamine and bacterial lipopolysaccharide proapoptotic actions in anterior pituitary cells [3]–[6], [18]. In fact, we observed that the apoptotic effect of 16 kDa PRL is also an estrogen-dependent process. Different mechanisms have been proposed by which estradiol sensitizes anterior pituitary cells to different apoptotic signals, including the expression of proapoptic Bax vs antiapoptotic Bcl-2 proteins [19] and TNF-α/TNFR1 and FasL/Fas systems [3], [4]. The increase in anterior pituitary N-terminal PRL fragments production observed at proestrus suggest that, among other factors, 16 kDa PRL is involved in apoptosis of lactotropes occuring at this stage of the estrous cycle.

In addition, we observed that 16 kDa PRL exerts an antiproliferative action in total anterior pituitary cells and lactotropes, regardless of the presence of estradiol. It has been described that 16 kDa PRL decreases the percentage of endothelial cells in S-phase, with a cell cycle arrest at G2/M transition and no concomitant increment of hypodiploid cells [11]. In our studies, 16 kDa PRL induced a decrease in the percentage of cells in S-phase independently of the presence of E2, whereas only in E2-treated cells 16 kDa PRL increased the percentage of hypodiploid cells. This finding leads us to speculate that during proestrus, the higher levels of 16 kDa PRL isoform induce a decrease in anterior pituitary cell proliferation, and due to the permissive action of estrogens present in that moment of the estrous cycle, the arrested cells die by apoptosis.

Besides its role in anterior pituitary physiology, some aspects of the biology of 16 kDa PRL can be related to the anterior pituitary under pathological conditions. Prolactinomas are the most common pituitary tumors which are usually non metastatic and non invasive. It is known that prolactinomas are less vascularized than normal pituitaries [30] and, due to their antiangiogenic activity, vasoinhibins were suggested to be, at least in part, responsible for this phenotype [31]. In addition, the antiproliferative and proapoptotic actions of 16 kDa PRL on anterior pituitary cells reported here, may contribute to the behavior of prolactin producing tumors.

In summary, our study indicates that the production of 16 kDa PRL by the anterior pituitary is variable along the estous cycle and increased by estrogens. In addition, we demonstrate the antiproliferative and estradiol-dependent proapoptotic effects of this vasoinhibin on anterior pituitary cells. These findings suggest that the action of estrogens on anterior pituitary cell homeostasis involves the modulation of vasoinhibins production and activity.
